# Development of an Internet of Things Technology Platform (the NEX System) to Support Older Adults to Live Independently: Protocol for a Development and Usability Study

**DOI:** 10.2196/35277

**Published:** 2022-05-05

**Authors:** Claire M Timon, Emma Heffernan, Sophia M Kilcullen, Hyowon Lee, Louise Hopper, Joe Quinn, David McDonald, Pamela Gallagher, Alan F Smeaton, Kieran Moran, Pamela Hussey, Catriona Murphy

**Affiliations:** 1 Centre for eIntegrated Care, School of Nursing, Psychotherapy and Community Health Dublin City University Dublin Ireland; 2 Insight Centre for Data Analytics Dublin City University Dublin Ireland; 3 School of Psychology Dublin City University Dublin Ireland; 4 School of Computing Dublin City University Dublin Ireland; 5 Davra Dublin Ireland; 6 Danalto Limited Dublin Ireland; 7 Insight Centre for Data Analytics, School of Health and Human Performance Dublin City University Dublin Ireland

**Keywords:** independent living, older adults, Internet of Things, wearable electronic devices, activities of daily living, mobile phone

## Abstract

**Background:**

In a rapidly aging population, new and efficient ways of providing health and social support to older adults are required that not only preserve independence but also maintain quality of life and safety.

**Objective:**

The NEX project aims to develop an integrated Internet of Things system coupled with artificial intelligence to offer unobtrusive health and wellness monitoring to support older adults living independently in their home environment. The primary objective of this study is to develop and evaluate the technical performance and user acceptability of *the NEX system*. The secondary objective is to apply machine learning algorithms to the data collected via the NEX system to identify and eventually predict changes in the routines of older adults in their own home environment.

**Methods:**

The NEX project commenced in December 2019 and is expected to be completed by August 2022. Mixed methods research (web-based surveys and focus groups) was conducted with 426 participants, including older adults (aged ≥60 years), family caregivers, health care professionals, and home care workers, to inform the development of the NEX system (phase 1). The primary outcome will be evaluated in 2 successive trials (the Friendly trial [phase 2] and the Action Research Cycle trial [phase 3]). The secondary objective will be explored in the Action Research Cycle trial (phase 3). For the Friendly trial, 7 older adult participants aged ≥60 years and living alone in their own homes for a 10-week period were enrolled. A total of 30 older adult participants aged ≥60 years and living alone in their own homes will be recruited for a 10-week data collection period (phase 3).

**Results:**

Phase 1 of the project (n=426) was completed in December 2020, and phase 2 (n=7 participants for a 10-week pilot study) was completed in September 2021. The expected completion date for the third project phase (30 participants for the 10-week usability study) is June 2022.

**Conclusions:**

The NEX project has considered the specific everyday needs of older adults and other stakeholders, which have contributed to the design of the integrated system. The innovation of the NEX system lies in the use of Internet of Things technologies and artificial intelligence to identify and predict changes in the routines of older adults. The findings of this project will contribute to the eHealth research agenda, focusing on the improvement of health care provision and patient support in home and community environments.

**International Registered Report Identifier (IRRID):**

DERR1-10.2196/35277

## Introduction

### Background

Globally, the population is aging. In Ireland, population estimates for 2021 revealed that adults aged ≥65 years accounted for 14.8% of the total population [1], and this percentage is expected to nearly double by 2051 [1]. In Ireland, we observed an increase in life expectancy, which surpassed the European Union average in 2017 because of significant reductions in major causes of death such as circulatory system diseases and cancer [2]. One of the challenges is that those who live longer do so with a combination of chronic disease, multi-morbidity, and frailty. Support for independent living is, therefore, a major concern for older adults themselves, in addition to family caregivers and health and social care providers. As the vast majority (95%) of older adults in Ireland live in private households [3] and want to remain living at home, this is primarily a community-based issue requiring a home-based response.

Connected health and remote health care are seen as key drivers of home-based health and social care delivery. The use of these technologies has the potential to sustain and accelerate improvements in quality of life and health, and enhance the independence of an aging population [4]. The emergence of new *smart home* and Internet of Things (IoT) technologies have the potential to integrate information technology with assistive technologies highlighting potential for ambient-assisted living systems. These assisted living systems have the potential to support an aging population to meet their needs with minimal digital literacy required [5]. Ambient-assisted living systems incorporate devices such as wearable and in-situ sensors, voice-controlled systems, and smartphones, all connected to the IoT that can be remotely monitored, controlled, or accessed and provide services that respond to the perceived needs of the users [6]. These devices generate and capture massive streaming data, which contain valuable information that needs to be mined to facilitate timely actions and better decision-making [7]. Machine learning and big data analytics will undoubtedly play a critical role in enabling the delivery of future smart care services. Machine learning and big data analytics have been investigated to facilitate the automatic recognition of activities of daily living (ADL) in older adult populations [8,9], which is an important component in the understanding of quality of life and health and well-being. Other data analytics approaches have focused on using periodicity intensity to identify deviations in day-to-day activity patterns in older adults [10].

Efforts to support independent living using IoT and wearable technologies among older adults have been investigated for a range of purposes, including physiological monitoring, for example, monitoring health status [11] notifying health care providers of changes in health status [12]; emergency detection and response, for example, falls detection [13]; safety monitoring and assistance, for example, reminding and prompting older people to take medication, which in turn supports independence and safety [14]; personal assistance, for example, automating daily tasks and home maintenance [15]; and social interaction monitoring and assistance, for example, enabling communication and connection with social networks [16]. However, evidence suggests that the use of wearables and IoT technologies to support independent living [5] highlights that a moderate to low usability or user-friendly approach is reported in most of the studies, largely owing to technical issues surrounding the deployment of technologies. Further research focusing on the usability and acceptability of IoT smart home systems in older adults is warranted.

The NEX project is a multidisciplinary research collaboration involving researchers at the Centre for e-Integrated Care, the School of Nursing, Psychotherapy and Community Health, the School of Psychology, the School of Health and Human Performance, and Insight Centre for Data Analytics in Dublin City University (DCU) working with leading Irish technology companies DAVRA and Danalto. This project was funded under the Disruptive Technologies Innovation Fund administered by Enterprise Ireland (grant DT-2018-0258). The overarching aim of the NEX project is to develop a technological solution that will enable older adults to remain living independently at home for longer periods and facilitate caregivers to care for their family members or clients or patients in a nonintrusive manner.

**Figure 1:**
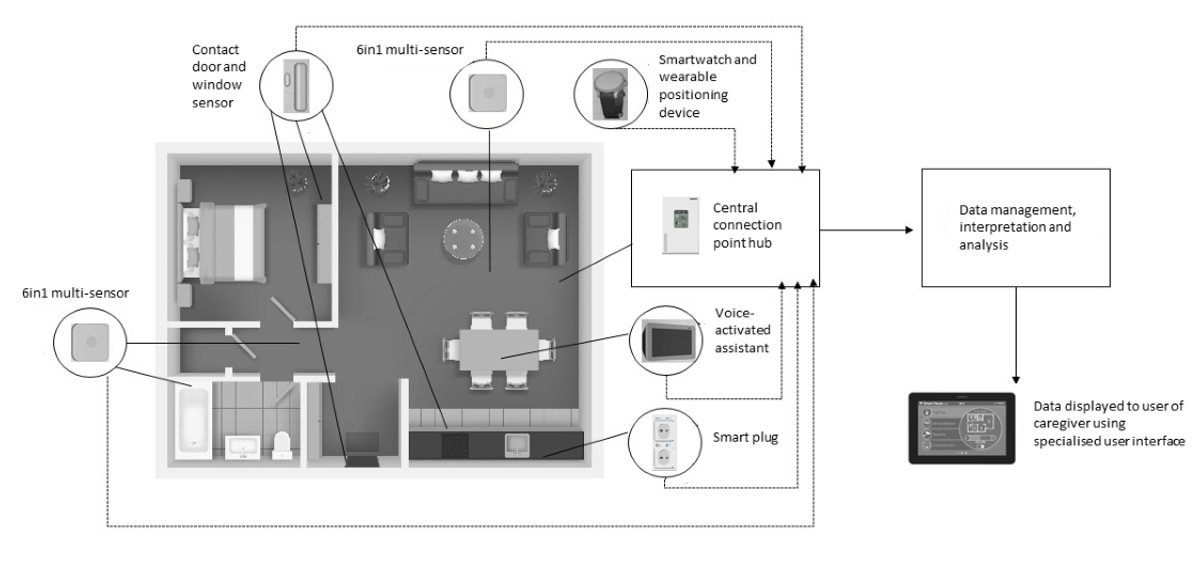
The NEX system.

The unobtrusive nature of the NEX system is critical to ensure that participants being monitored barely notice the existence of the sensing device or procedure, and therefore, are prone to less bias and burden. Codesign approaches with relevant stakeholders (described in phase 1) inform the design of the NEX system. The final NEX system design consists of a range of IoT technologies, including a smartwatch (measurement of sleep and step count), voice-activated assistant (entertainment and reminder functionality), contact sensors (detect activity around the home and opening and closing of doors and cupboards), smart plugs (measure energy use of appliances), motion sensors (detect movement, temperature, humidity, and light in the home), positioning wearable device (developed by industry partner Danalto to distinguish the activity of the user in multi-occupancy households), hub (central connection point for sensor devices), tablet (display NEX system data to participants), and a cloud-hosted secure device management (developed by the industry partner DAVRA), as shown in Figure 1. Innovation in the NEX system is underpinned by the use of a variety of IoT technologies in conjunction with artificial intelligence to develop an integrated system that can detect changes in the usual routine (periodicity and ADL detection). The data generated from the IoT devices will be used to drive the automatic detection of ADLs that are presented back to caregivers and participants. The rules for detecting these ADLs will, in turn, be automatically created using the well-known a priori algorithm for mining association rules [17]. In addition, a positioning device for the purpose of personalizing activities within the home (will assign an action; eg, use of a door or an ambient value such as motion to a specific individual within a household) is being developed as part of this project to support the integrated system.

The data generated from the NEX system may objectively depict aspects of health and behavior affected by age-related changes, and the data generated from this system could serve as an insightful resource when making health-related decisions. For example, sedentary lifestyle is a well-known age-related change prevalent among many older adults and has been associated with adverse health [18]. Previous studies have highlighted that, in some cases, self-report activity data do not accurately reflect actual activity levels [19] and therefore have limited use in providing insights required to make positive changes. The NEX system, which incorporates wearable sensors and other types of sensing devices with advanced data analytics, has the potential to objectively identify ADLs and provide a detailed analysis of users’ activity levels and lifestyles. This in turn would allow older adults to view their data and identify the specific context of how sedentary behaviors occur (eg, reading a book and using a computer). The NEX system data can be used to consider adaptive strategies; for example, the use of timely reminders to promote physical activity. By having access to their detailed health data and behavioral patterns, older adults can make timely adjustments in health behaviors themselves or present the data to health care providers and family caregivers to develop personalized adaptive strategies together. Equally, the impact of these strategies or interventions can be objectively monitored in the home environment, thus providing feedback to older adults and care providers on outcomes.

### Objective

The project was structured in 3 phases to achieve the overall project aim. Phase 1 aims to work with stakeholder groups (older adults, health care professionals, family caregivers, and homecare support workers) to identify user needs and requirements to inform the NEX system design using mixed methods research. Phase 2 aims to investigate the technical performance and understand the participant engagement of the NEX system as a pilot testing phase for a duration of 10 weeks. Phase 3 of the project will commence in January 2022, and will incorporate the findings from phase 2 to inform the refined NEX system design. This phase will focus on a larger-scale, real-world deployment of the NEX system (n=30 older adults aged ≥60 years) being tested in the homes of participants for a minimum of 12 weeks. At the end of the 12-week period, participants can decide whether they would like to use the NEX system for a further period. A longer timespan is needed for the trial in phase 3 so that the periodicity of habits associated with normal living can be established. In addition to investigating the technical performance of the system in real-world deployment, a key research objective of phase 3 is to develop an automated approach to identify ADL unique to each participant. This study aims to describe the 3 phases of the overall NEX project.

## Methods

### Phase I: User Needs Requirements

#### Study Overview

Despite mounting evidence of the role of technology in supporting older adults to live independently at home [5,20,21], it is critically important that any proposed technology be aligned with core requirements. This underpins the objective of this phase: to provide user needs and requirements to ensure the development of systems that blend user needs with technological advancements. A user-centered design approach [22] was used to identify user needs and requirements. This codesign approach focused on partnering with end users to design the NEX system technology. The aim of the codesign workshops was for older adult participants and other stakeholder groups (family caregivers, home support workers, and health care professionals) to play an active role in the exploration of their needs and the possibilities that technology can bring to their lives.

#### Participants

Stakeholder recruitment took place in January 2020, and initial codesign face-to-face workshops commenced in March 2020. The COVID-19 pandemic and subsequent restrictions on movement resulted in a change in methodology to facilitate data collection during this period. To develop the project plan, the research team designed and deployed a web-based survey in June 2020 and conducted web-based workshops from August to October 2020. In all, a total of 4 stakeholder groups were recruited for the user needs requirements (UNR) aligning with the target population; these included older adults, family caregivers, home support workers and health care professionals. Overall, 426 stakeholders participated in the face-to-face workshops, web-based surveys, and web-based workshops. Older adult stakeholders were the largest participant group (265/426, 62.2%), followed by family caregivers (83/426, 19.5%), health care professionals (51/426, 12%), and home care support workers (27/426, 6.3%).

#### Procedures

Using a user-centered participatory codesign methodology, face-to-face workshops were conducted, including the use of personas designed to be fictitious but authentic end users. Participants assisted in developing scenarios around the persona that captured a particular issue, where technology could enhance self-management competencies or provide the support needed. A total of 2 workshops involving 17 participants (15 older adults and 2 family caregivers) were completed in March 2020 before the introduction of the first set of restrictions associated with COVID-19.

The onset of the COVID-19 pandemic resulted in an adjustment to our methodology, including a web-based survey and web-based workshops. The overall aim of the web-based survey was to gain insights from the 4 key stakeholder groups to examine how technology might play a greater role in supporting older adults to live independently at home to assist in the development of the NEX system. One overarching survey was designed as 4 individual surveys with specific questions tailored to each stakeholder type. One participant classification question assigned each participant to a relevant set of survey questions. In brief, the survey consisted of a mixture of open-ended (free-text response questions) and forced-response single- and multiple-choice option questions.

Following the web-based survey, 13 web-based workshops were conducted to facilitate discussions centered on stakeholders’ opinions on how technology can support independent living and to gather user views about specific forms of technology to support independent living for older adults. Specific technology solutions (voice-activated assistant, ambient sensors, wearables, and an integrated system) were demonstrated to the participants via video. Survey responses were exported to SPSS (version 25; IBM Corp), and descriptive statistics and chi-square analyses were applied to examine common attitudes among the respondents. Inductive thematic analysis [23] was performed on free-text responses from the survey and all workshop transcripts.

The research efforts described above highlight several key user requirements, design, and implementation considerations. The research team developed a user needs requirement specification document outlining the UNR, the potential system requirements (the functions of the system must be able to perform), the data requirements (the types of information that a system must be able to process), and other requirements, including user support and training. This enabled the research and technology partners to map user needs to technology solutions while ensuring that the user, system, and data requirements were adhered to.

### Phase 2: Friendly Trial

#### Study Overview

With the UNR study (phase 1) phase of the project, the primary objective of the phase 2—Friendly trial was to investigate the technical performance and participant engagement of the proposed NEX system as a pilot for phase 3. To achieve this, the proposed system was installed in the homes of 7 healthy older adults (aged ≥60 years) for 10 weeks. The proposed NEX system consists of a voice-activated assistant, ambient sensors, and a wearable accelerometer-based device. Consumer devices are used in the NEX system prototype to ensure safety, reliability, and acceptability. An external market analysis was completed, and the devices highlighted in Textbox 1 were selected on the basis that these devices had an excellent connection process, long-lasting battery life, ease of installation, and most importantly, these devices did not require a dedicated router in the participants’ homes, which reduced the amount of technology that needed to be installed in the participants’ homes. All technology providers were screened by the DAVRA (industry partner) compliance team to ensure that the devices were International Organization for Standardization 27001 certified (an internationally recognized certification of data security) and were compliant with the General Data Protection Regulation and the Health Insurance Portability and Accountability Act. In addition, a Data Protection Impact Assessment was completed by the DCU to ensure the safety and privacy of all the trial participants’ data.

Before the Friendly trial, the original plan was to demonstrate and test the system in the DCU community laboratory environment with participants to identify and resolve issues relating to the NEX system, which could be addressed before the technology was installed in participants’ homes. However, COVID-19 pandemic restrictions impacted the ability to bring participants into the community laboratory in the DCU and the ability of technicians and researchers to visit participants’ homes for installation and training. To overcome these barriers, the Friendly trial was redesigned to involve the self-installation of the NEX system by the participants themselves with remote support. To investigate the feasibility of this self-installation for the Friendly trial, 2 researchers conducted self-installation with remote support in their own homes. The NEX system was installed at each home for approximately 12 weeks. The NEX system consisted of (Textbox 1) contact sensors on entry and exit doors to home and contact sensors on drawers and cupboards in the kitchen; smart plugs for kitchen appliances; 6-in-1 sensors to detect motion within rooms in the home alongside temperature, humidity, luminescence, UV light, and vibration; a Sony mWatch as an alert system (call for assistance) with GPS tracking and for measurement of sleep duration and step count; and an Amazon Echo Show 8 voice-activated assistant for entertainment and reminder functionality. The researchers’ demonstrator efforts highlighted key issues related to greater preconfiguration of the system, more installation and training support, ongoing technology management (eg, charging), and more instruction regarding the removal of the components of the NEX system. It also highlights that the proposed NEX system may be burdensome for self-installation in the target group of older adults. This feedback was communicated to the technology partners and resulted in the production of high-quality installation manuals and videos and a 24-hour technical support helpline to support the Friendly trial self-installation.

**The technology components of the Friendly trial NEX system.**

**Friendly trial NEX system components**

- Sony mWatch
- Amazon Echo show 8
- Samsung SmartThings hub
- Aeotec door and window sensor 6
- Samsung SmartThings smart plugs

#### Participants

In May 2021, 7 healthy older adult participants were recruited to the NEX Friendly trial, whereby the NEX system was installed by participants themselves in their own homes for a duration of 10 weeks. For the purposes of this research, *healthy older adults* were defined as adults aged ≥60 years living independently with or without one or more stable chronic conditions. Older adults who are currently acutely ill or who meet any of the criteria for *very high risk* with relation to COVID-19 [24], apart from age, were not eligible to take part. The aim of this study is to investigate the performance and participant engagement of the potential components of the *NEX system*. Participants (N=7) ranged from 63 to 87 years of age; 5 (71%) participants were women, and the remaining 2 (29%) were men. All participants were living alone in urban areas of Ireland (6 living in Dublin [urban] and 1 living in a rural location outside Dublin). Although most participants (6/7, 86%) described their health as “very good” or “excellent,” 86% (6/7) of participants reported having one or more chronic illness and 44% (3/7) of participants noted that physical modifications were made to their home to assist with access. All participants had a home broadband connection, reported regular use of a smartphone, indicated some level of familiarity with technology, and were willing to install the system themselves with remote support.

#### Procedures

This version of the NEX system consisted of a voice-activated assistant, ambient sensors (motion and contact sensors), and a wearable accelerometer-based watch device. All aspects of the trial and interactions between the participants and the research team took place remotely over Zoom and via email and phone. The Friendly trial finished in September 2021, and the data analysis will be finalized by March 2022. The investigators will assess the technical performance of the NEX system by analyzing aspects of front-end usability (examining bugs and number of crashes, etc) and back-end issues (eg, memory and database integrity). Lighthouse analytics will be used for technology performance statistics. Participant engagement was also investigated by analyzing the data collected from the technologies to identify usual behavior patterns over time (periodicity). Data modeling based on a sliding window and association rule mining was completed with dietary intake data reported by participants, and sensor and smart plug data from their kitchens to identify eating occasions. Participants also completed a process evaluation interview and questionnaires (adapted version of the Technology Acceptance Model) [25] and System Usability Scale [26] to assess the acceptability and usability of technology devices. Process evaluation interviews (questions based on the Theoretical Domains Framework of behavior change [27]) were transcribed, and thematic analysis [23] was performed.

### Phase 3: The Action Research Cycle

#### Study Overview

The final phase of the overall NEX project aims to demonstrate a refined NEX system working in a home environment. The first objective of this study is to use the NEX system to collect data for the automatic identification of patterns of typical behavior (periodicity) for the identification of ADLs. ADLs are essential and routine tasks that most healthy individuals can perform without assistance [28]. The inability to accomplish essential ADL may lead to unsafe conditions and poor quality of life and may indicate a physical or cognitive disability in older adults [29]. Eligibility for home care is frequently associated with deficits in ADL ability [30,31]. Assessment of ADLs through self-reported, observed, or objective data provides evidence to individuals and caregivers of deficits in self-care ability and supports potential interventions that may be required for continued independence [32]. The investigators aim to focus on using sensors and smart plugs to facilitate the identification of the following ADLs: (1) eating and drinking events, (2) dressing, (3) bathing or showering, (4) getting up from and going to bed, (5) activity around the house, and (6) time spent outside the house.

A secondary research objective is to investigate the participants’ engagement with the system by completing interviews with participants about their experience of using the NEX system and about system acceptability and usability. From a person-centered and ethical viewpoint, it is expected that NEX technology will have a positive impact on older users’ lives. Therefore, it is valuable to investigate user psychological factors such as current mental well-being, satisfaction with current levels of daily novelty and participation in meaningful activity, and levels of general self-efficacy not only to facilitate building richer user profiles for technology developers but also to facilitate the exploration of the extent to which this technology may affect (positively or otherwise) these important life areas. Finally, the third research objective of this study is to recruit caregivers (n=5) and present caregiver participants with NEX system data to investigate the perceived usefulness of assisting with the provision of care to older adults.

#### Participants

For the purposes of this research trial, the investigators will aim to recruit a target sample size of n=30 (the sample size is based on population sizes from other published studies in this area) [33,34]. healthy older adults (aged ≥60 years) who live independently at home in the community. All participants must have the capacity to provide consent and be willing to provide informed consent to participate. For the purposes of this research, *healthy older adults* are defined as adults aged ≥60 years, living independently with or without one or more stable chronic conditions. In addition to testing a refined NEX system in this trial, the investigators aim to develop a new technology that will distinguish sensor interactions in households with frequent visitors. Participant recruitment commenced in November 2021, with an anticipated start date for the Action Research Cycle trial of January 2022.

#### Procedures

Eligible participants will enroll in the Action Research Cycle study for a minimum of 12 weeks, which will involve a mixture of in-home visits with a NEX project researcher and technician, and study visits will be conducted via Zoom. During the first visit, participants will complete an informed consent form, a demographics questionnaire, a questionnaire about technology use, and a compilation of health and well-being assessments. These assessments include health-related quality of life (EQ-5D-5L) [35], frailty assessment (Program of Research to Integrate Services for the Maintenance of Autonomy—PRISMA [Program of Research on the Integration of Services for the Maintenance of Autonomy] 7) [36], and Minicog (assessing memory and cognitive function) assessment [37]. In addition, the researchers (CMT, EH, and SK) will complete the following questionnaires: Novelty Need Satisfaction Scale [38], General Self-Efficacy Scale [39], Preference for Routine Scale [40], Meaningful Activity Participation Assessment [41] and Warwick-Edinburgh Mental Well-being Scale [42]. These measures, which have been previously used in other studies involving older adults [38,40-43], will facilitate an exploration of the extent to which the NEX system addresses these diverse needs. All assessments will be completed by a researcher in person or over a Zoom call where possible. At the end of this visit, the researcher will ask the participant for their Wi-Fi name and password so that all technologies can be preconfigured and paired to their network before installation in visit 2.

During the second visit, a researcher and technical engineer visited the participant in their home environment to facilitate the installation of the NEX system technology. The researcher and technician will complete a home configuration assessment with the participant by identifying the most appropriate locations to install NEX system technology. The technology that is integrated to form the NEX system is listed in Textbox 2, and an overview of the final system design is depicted in Figure 1.

**The technology components of the Action Research Cycle trial NEX system.**

**Action Research Cycle trial NEX system components**

- Withings Smartwatch
- Amazon Echo show 8
- Aeotec hub
- Aeotec door and window sensor 7
- Aeotec multisensory 6
- Aeotec Smart Switch 6 (smart plugs)
- Lenovo smart tablet M8
- Danalto positioning wearable device with associated hubs
- Cloud-hosted secure device management, identity, and activities of daily living analytic engines

The above technologies will be deployed in combination to facilitate the detection of some of the key ADL from the participants’ sensor, wearable, and smart plug use data over the trial period. The system will be installed in the homes of the participants by a technical engineer from DAVRA during this visit. Training on the technology will be provided to the participants at the time of installation, and a training manual will be provided. Specifically, participants will be shown how to use their step count and sleep data via an app on the tablet provided, as well as how to use the entertainment functionality of Alexa Echo Show 8, for example, listen to the radio or ask questions, and how to set reminders, for example, to take medication. A dedicated NEX mobile helpline will be set up so that the participants can contact the research team with any problems at any time during the trial. The technical engineer will also be available via a technical helpline to consult or visit the participant if any of the technologies stop working. The participants are free to interact with the technology as little or as often as they wish during the trial period. Over the course of the trial, a NEX researcher will meet with the participants over Zoom on 4 separate occasions. During these interactions, participants (and their respective caregivers for n=5 participants) will be trained on how to interpret the data collected from the NEX system installed in their homes. Caregiver participants will be presented with a sample of raw data and ADL data collected from the technology installed in the homes of the older adults to whom they provide care. Caregivers will be interviewed about their perceptions of these data and the perceived usefulness of having access to these data when providing care and support. The research team will also investigate the perceived usefulness of these data for older adult participants trailing the NEX system in their homes. In addition, the researchers will gather ground truth data to validate the ADL data recorded via the technology; for example, What time did you get up from bed yesterday morning? During the final visit, the researcher will interview the participants about their experience of the trial, their experience and perception of each technology, and the NEX system as a whole, and complete an assessment of the system acceptability and usability (adapted version of the Technology Acceptance Model [25] and System Usability Scale [26]). In addition to the data collected as part of these questionnaires, the research team will consider the overall use and interaction with the NEX system technology; for example, smart plug use data and wearable devices, from the data collected over the trial period to understand the overall usability and acceptance of the system. The researcher will also repeat the EQ-5D-5L [33], Novelty Need Satisfaction Scale [38], General Self-Efficacy Scale [39], Preference for Routine Scale [40], Meaningful Activity Participation Assessment [41], and Warwick-Edinburgh Mental Well-being Scale [42] to investigate whether having NEX installed in participants’ homes for the duration of the trial affects their quality of life and other aspects of life. The NEX system will subsequently be removed from the participants’ homes by the NEX project technical engineer.

### Ethics Approval

Participants in phases 1 and 2 were recruited via older adults, family caregivers, and health care professional organizations in Ireland. Participants were also recruited via DCU’s Age Friendly University network, local council age friendly offices, and social media campaigns on Facebook and Twitter. Recruitment for phase 3 will follow a strategy similar to phase 2. Phases 1, 2, and 3 of the projects are not considered to have exposed participants to danger or discomfort. As phases 2 and 3 relate to the installation of technologies in the homes of older adults, there is a low risk that participants may become distressed or overwhelmed by this experience. To mitigate this, a support phone line was established, and regular communication between the participants and the research is a core feature of the project. In the unlikely event that participants become distressed, the research team will discuss the difficulty with the participant and help them reflect on the best course of immediate action. This may include taking a break from the trial, having the technology uninstalled from their homes, discontinuing participation, or, if necessary, with their consent, referring them to the project principal investigator. The investigators have explicitly highlighted to the DCU ethics committee and to participants that the NEX system data will not be monitored in real time; therefore, no intervention will be performed. If the researcher or participant has any concerns related to these questionnaires and assessments, the results will be sensitively discussed with participants, and they will be encouraged to speak to their general practitioner. Participation is voluntary, and oral and written information will be provided to participants regarding the purpose of the study and how their data will be used in the research. Ethical approval to conduct this study was obtained from the DCU Research Ethics Committee for phase 1 (DCUREC2019223), phase 2 (DCUREC2020180), and phase 3 (DCUREC202221).

## Results

The project is funded for a 3-year period (2019-2022), and enrollment for phase 1 was completed in 2020. In phase 1, 426 participants (older adults, family caregivers, and health care professionals) were recruited to participate in research activities (face-to-face focus groups, web-based surveys, and web-based focus groups) to identify user needs and requirements to inform the development of the NEX system. Phase 2, which focused on pilot testing of the initial version of the NEX system, was completed in September 2021. A total of 6 older adult participants installed the NEX system technology in their home environments with remote support and completed a 10-week trial. Although the small sample size of participants makes it difficult to generalize the acceptability results, the technical evaluation and feedback from the Friendly trial will be used to refine the design of the NEX system for larger-scale deployment in the homes of 30 older adult participants (phase 3), which is expected to be completed by June 2022. Ethical approval for phase 3 was granted in November 2021, and the first results (phase 1) are expected to be submitted for publication in March 2022.

## Discussion

### Principal Findings

The NEX project brings together citizens, partners from the fields of health, technology, data analytics, and industry to develop timely technological solutions to support older adults living independently. The findings of phases 1 and 2 have informed the design of the NEX system, which has facilitated the collection of rich and extensive health and activity data from users’ home environments. It is anticipated that the findings of phase 3 will indicate the potential usefulness of the ADL detection and periodicity data approach for facilitating future IoT interventions in-home and community environments.

Although positive advances have been made in this area of research, there are significant challenges related to the usability and acceptability of IoT technologies for supporting independent living [5]. Specific issues related to the deployment of the technologies; that is, inaccurate sensors, battery or power issues, restricting users within the monitoring area, and lack of interoperability. In addition, the lack of user-centered design approaches in the development phases may also contribute to the low usability observed in some instances [5]. A particular strength of this study relates to the emphasis on user-centered design approaches involving multiple key stakeholder groups in phase 1. However, it remains to be seen whether the acceptability of the NEX system and the accuracy of the sensor data collected will be impacted by the technical issues described above.

An important consideration for the future development of NEX systems relates to how to deliver the data generated by the NEX system in an intuitive and easy-to-interpret manner. This is necessary to ensure that older adults can easily access their data to facilitate insight into their ADLs. Self-awareness of age-related changes and how they impact ADLs in a personal context is especially important for developing appropriate strategies to address these changes [44].

### Conclusions

Although further research is warranted, it is anticipated that the findings of the NEX project will contribute to the design and development of future robust studies involving the trailing of IoT technologies in-home environments for older adults.

## Abbreviations

ADL: activities of daily living
DCU: Dublin City University
IoT: Internet of Things
PRISMA: Program of Research on the Integration of Services for the Maintenance of Autonomy
UNR: user needs requirements
